# Reuse of Textile Dyeing Effluents Treated with Coupled Nanofiltration and Electrochemical Processes

**DOI:** 10.3390/ma9060490

**Published:** 2016-06-20

**Authors:** Valentina Buscio, María García-Jiménez, Mercè Vilaseca, Victor López-Grimau, Martí Crespi, Carmen Gutiérrez-Bouzán

**Affiliations:** 1 Institute of Textile Research and Industrial Cooperation of Terrassa (INTEXTER), Universitat Politècnica de Catalunya-BarcelonaTech (UPC), C/Colom 15, Terrassa 08222, Spain; valentina.buscio@intexter.upc.edu (V.B.); graci.927@hotmail.com (M.G.-J.); vilaseca@intexter.upc.edu (M.V.); victor.lopez-grimau@upc.edu (V.L.-G.); crespi@etp.upc.edu (M.C.); 2 Department of Project and Construction Engineering, Universitat Politècnica de Catalunya-Barcelona Tech (UPC), C/Colom 11, Terrassa 08222, Spain

**Keywords:** dyeing effluent, nanofiltration, electrochemical process, water reuse

## Abstract

The reactive dye Cibacron Yellow S-3R was selected to evaluate the feasibility of combining nanofiltration membranes with electrochemical processes to treat textile wastewater. Synthetic dyeing effluents were treated by means of two nanofiltration membranes, Hydracore10 and Hydracore50. Up to 98% of dye removal was achieved. The influence of salt concentration and pH on membrane treatment was studied. The best dye removal yield was achieved at pH 3 in the presence of 60 g/L of NaCl. After the membrane filtration, the concentrate containing high dye concentration was treated by means of an electrochemical process at three different current densities: 33, 83, and 166 mA/cm^2^. Results showed a lineal relationship between treatment time and applied current density. Both permeates and electrochemically-decoloured effluents were reused in new dyeing processes (100% of permeate and 70% of decoloured concentrates). Dyed fabrics were evaluated with respect to original dyeing. Colour differences were found to be into the acceptance range.

## 1. Introduction

One of the biggest problems of the 21st century is the quality and management of water. Although the water is the most abundant element on Earth, only a small percentage (about 0.3%) can be used by humans [1]. In 2014, according to the Food and Agriculture Organization of the United Nations (FAO), industrial water withdrawals account for 19% of global water demand [2] and an increase up to 22% is expected for 2030 [3]. The increase in water consumption and consequently the increase of water scarcity have caused the creation of new environmental policies focused on recycling and reuse of water.

Textile industry is one of the largest consumers of water due especially to their finishing processes such as dyeing and subsequent washing steps. In addition to high water consumption, the textile industry is also characterized by the generation of huge amounts of wastewater. This wastewater contains different kinds of dyes and chemical additives [4,5], which cannot be easily degraded.

Nowadays, biological [6,7,8] and physical-chemical processes [9,10,11] are used to remove dyes from textile wastewater. Biological processes show high efficiency in organic matter removal (about 90%) [12], but low dye degradation due to the poor biodegradability of dyes. Physicochemical processes such as coagulation-flocculation enable to decolourise completely the effluent [13] but generate a concentrate that must be treated. It is important to highlight that none of them enables water reuse in textile processes [14,15]. On the other hand, treatments such as photo-Fenton [16] and photocatalytic [17,18] have been also studied. These methods provided high colour removal (between 90% and 98%) but the high treatment cost is their main limitation [19].

Membrane processes can be applied to remove different kind of dyes. In addition, they produce a high quality permeate which can be reused in new textile processes [20]. In some cases, they also allow both the reuse of auxiliary chemicals and some concentrate dyes [21]. Taking into account their significant advantages with respect to the others treatments, several authors have studied different membranes such as ultrafiltration (UF) [22,23,24], nanofiltration (NF) [25,26,27], and reverse osmosis (RO) [28,29] to treat textile wastewater. Nanofiltration membranes achieve almost 98% of dye rejection, whereas UF has shown to be able to remove about 90% of dye [30]. In terms of operating pressure and fouling, both UF and NF show fewer limitations than RO membranes [25].

Electrochemical processes have also been tested to remove dyes from textile effluents. The process is based on the direct degradation of dye on the anode using chloride as electrolyte [31] and on the indirect oxidation of dyes using the generated species. Dyeing processes with reactive dyes require high amounts of chloride to fix the dye on the fibre. Therefore, wastewater from this process is suitable to be treated by means of electrochemical treatment without further electrolytes or salt addition [32].

Taking these considerations into account, the aim of this work is to study the feasibility of combining nanofiltration membranes and electrochemical processes to treat textile wastewater containing a tri-reactive dye. For the membrane filtration study, the effect of salt concentration and pH of the effluents was tested. Subsequently, the concentrate obtained in the membrane filtration was decolourised by electrochemical treatment at three current densities. Both permeate and effluent decolourised by electrochemical process were reused in new cotton dyeings. Finally, fabrics dyed with the reused effluent were compared with fabrics dyed with softened tap water.

## 2. Materials and Methods

### 2.1. Reagents

**Figure 1:**
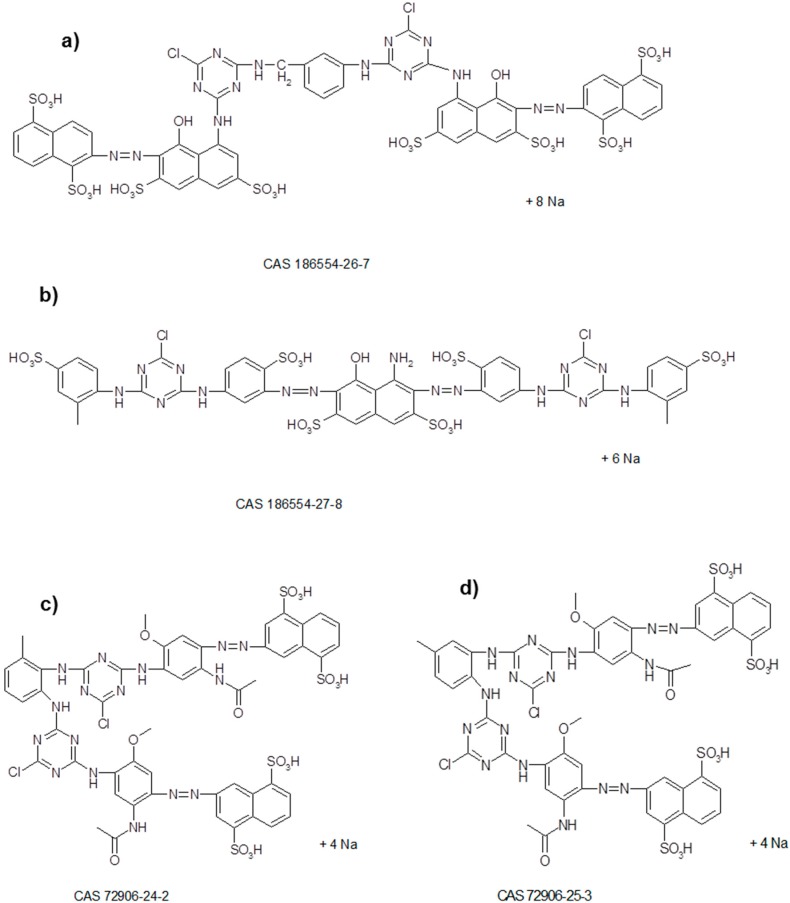
Chemical structure and CAS number of the dyes: PC (**a**); PN (mixture of **a**,**b**); PY (mixture of **c**,**d**).

The tri-reactive dye Cibacron Yellow S-3R (CY) was selected for this study. Three reactive dyes were selected for the study of water reuse: Procion Yellow H-EXL (PY), Procion Crimson H-EXL (PC), and Procion Navy H-EXL (PN). All dyes were provided by DyStar (L’Hospitalet de Llobregat, Spain). Figure 1 shows the chemical structures corresponding to PY, PC, and PN. The formula corresponding to CY has not yet been published.

Industrial effluents were simulated using sodium carbonate obtained from Sigma-Aldrich (Madrid, Spain) and sodium chloride purchased from Scharlau (Sentmenat, Spain). To test the effect of pH on the nanofiltration process, NaOH and HCl supplied by Scharlau (Sentmenat, Spain) were used.

The detergent COTEMOLL TLTR supplied by Color Center (Terrassa, Spain) was used in the washing step of the dyeing process. 

Sodium hypochlorite solution (6%–14% active chlorine) acquired from Sigma-Aldrich (Madrid, Spain) was used for the membrane cleaning.

### 2.2. Reactive Synthetic Effluent Preparation

Synthetic effluents of CY were prepared in softened tap water. Sodium carbonate (16 g·L^−1^) and sodium chloride (60 g·L^−1^) were added to the solution to simulate the pH and conductivity of industrial effluents.

### 2.3. Membrane Treatment

Two nanofiltration membranes, Hydracore50 (H50) and Hydracore10 (H10), provided by Hydranautics (Oceanside, CA, USA), were selected for this study. Their main characteristics are shown in Table 1.

**Figure 2:**
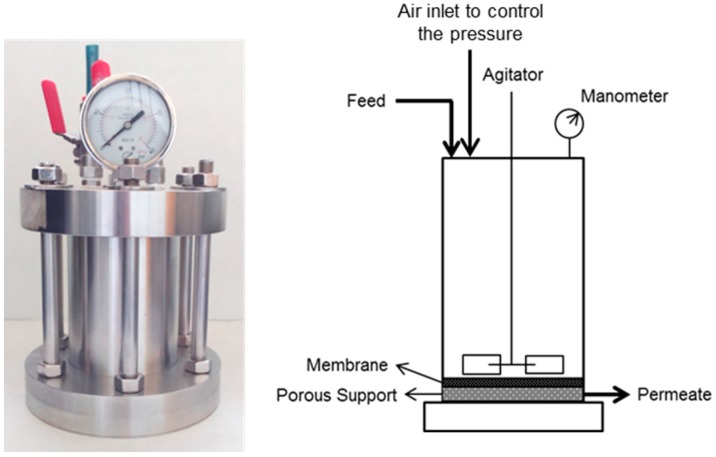
Membrane pilot plant.

A laboratory pilot plant (*V* = 0.5 L) manufactured by Polymem Company (Toulouse, France) was used in this work. The pilot plant operated in batch mode at room temperature. The pressure was maintained constant at 8 bars by manually operating a valve. The tests were carried out in dead-end filtration with agitation. The area of the membranes was 0.0064 m^2^ and it was placed over a plastic porous support. Figure 2 shows the scheme of the membrane pilot plant.

Filtration experiments were performed with 0.4 L of synthetic effluent (manually introduced into the cell) and conducted until 0.3 L of permeate were collected. A longer test (up to 2 L of permeate) was also carried out with the H10 membrane to observe its behaviour. In this case, the permeate flux was determined after every 0.4 L of permeate were collected.

After each experiment, a cleaning process was carried out. Membranes underwent the following steps:

- Soaking in deionized water for 10 min. This step was repeated three times;
- Membrane was immersed in a solution containing 0.005 g·L^−1^ active chlorine overnight;
- Rinsing with deionized water; and
- Soaking in deionized water for 10 min. This step was repeated three times.

### 2.4. Electrochemical Treatment

Electrochemical treatments were carried out in batch mode using an undividable electrolytic cell equipped with cylindrical electrodes made of Ti/Pt. The pilot plant was also equipped with a stirrer at 300 rpm. The active surface of each electrode was 0.006 m^2^. The experiments were performed with 2 L of synthetic effluent. The concentrates, containing 1 g·L^−1^ reactive dye, were treated at three current densities: 33, 83, and 166.6 mA/cm^2^. The voltage was variable between 4.5 and 6.3 V, depending on the current applied.

**Figure 3:**
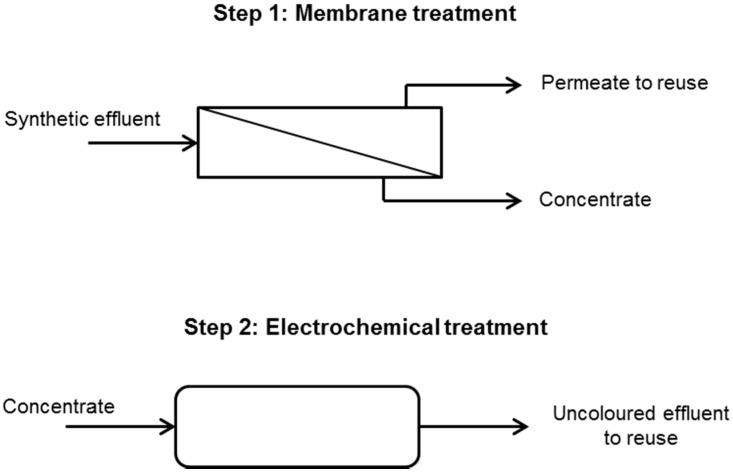
Combination of the nanofiltration and electrochemical process scheme.

To sum up, the entire treatment proposed in this work is shown in Figure 3.

### 2.5. Effluent Reuse

The reuse of the obtained permeates and uncoloured effluents after the treatment of synthetic effluents containing CY was studied with the four reactive dyes selected.

**Figure 4:**
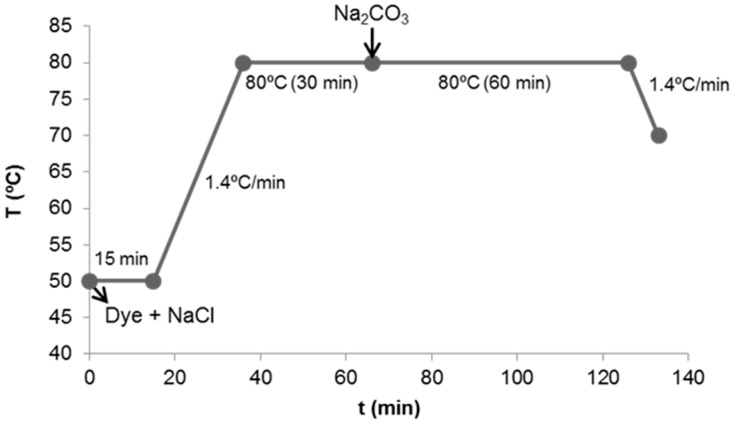
Dyeing procedure.

The reuse dyeing tests were performed in a laboratory Ti-Color dyeing machine (Prato, Italy) under the following conditions: 10 g of cotton fabric, dye concentration of 3% o.w.f (over weight of fibre), liquor ratio 1:10 (1 g fibre/0.01 L dye bath), 60 g·L^−1^ of NaCl, and 16 g·L^−1^ of Na_2_CO_3_. The dyeing procedure is shown in Figure 4.

After the dyeing process, a washing process was carried out. This process consists of nine steps:

- 1st–3rd: Cleaning with softened tap water at 50 °C for 10 min;
- 4th: Soap cleaning with 2 g·L^−1^ COTEMOLL TLTR at 95 °C for 15 min;
- 5th: Cleaning with softened tap water at 50 °C for 10 min;
- 6th: Soap cleaning with 2 g·L^−1^ COTEMOLL TLTR at 95 °C for 15 min; and
- 7th–9th: Cleaning with softened tap water at 50 °C for 10 min.

All of the experiments were run in duplicate and performed at a liquor ratio 1:10.

### 2.6. Analytical Methods and Measurements

The permeate flux was determined to evaluate the membrane fouling. It was determined by measuring the permeate volume collected in a certain period and using the following equation:

*J* = *V*/(*A* × ∆*t*) (1)

where *J* is the permeate flux (L·m^−2^·h^−1^), *A* is the effective area of the membrane (m^2^), and *V* is the collected volume in a time interval ∆*t* (L·h^−1^).

Dye removal (%*R*_dye_) was calculated from the initial concentration (*c*_i_) and dye concentrations at time t (*c*_t_) using the following equation:

*R*_dye_ = [(*c*_i_ − *c*_t_)/*c*_i_] × 100 (2)

A Shimadzu UV-VIS spectrophotometer UV-2401 (Kyoto, Japan) was used for dye absorbance measurements at the maximum wavelength of the visible spectrum. Dye concentrations were calculated according to the equation:

Abs = 20.447 × conc − 0.0058 (*R*^2^ = 0.9994); λ = 416 nm

The quality of dyed fabrics was determined in conformity with the Standard UNE-EN ISO 105-J03 [33]. Total colour differences (DE_CMC(l:c)_) were calculated from lightness (DL*), chroma (DC*), and Hue (DH*) using the following equation:

DE_CMC(l:c)_ = [(DL*/lSL)^2^ + (DC*ab/cSc)^2^ + (DH*ab/SH)^2^]^1/2^ (3)

For these measurements, a MINOLTA CM 3600d spectrophotometer (Osaka, Japan) was used. The measurements were performed with the standard illuminant D65/10°.

In general, a dyeing is considered to be in the acceptable range when the DE_CMC(l:c)_ value, with respect to a reference sample, is lower than 1.5.

## 3. Results and Discussion

### 3.1. Membrane Treatment

The synthetic dye baths containing 0.1 g·L^−1^ of CY dye were treated by means of a nanofiltration membrane laboratory pilot plant until 0.3 L of permeate was collected. In all of the experiments, the permeate flux remained almost constant: 28.4 L·h^−1^·m^−2^ for the H50 membrane and 37.5 L·h^−1^·m^−2^ for the H10 membrane.

**Figure 5:**
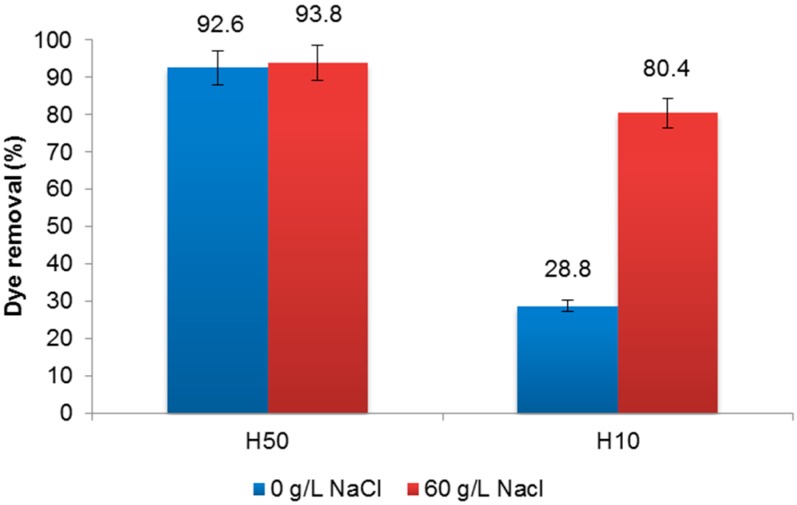
Effect of NaCl concentration on CY dye removal by a nanofiltration membrane.

To test the effect of NaCl on dye removal, experiments were carried out without salt and containing 60 g·L^−1^ of NaCl. According to results showed in Figure 5, the presence of NaCl did not affect the retention of the dye with the H50 membrane. However, in the case of the H10 membrane, a significant increase in dye removal was observed in the presence of salt. A further study may be required to determine if the NaCl increases the affinity between dye and membrane and/or causes agglomerations of dye particles large enough to be retained.

**Figure 6:**
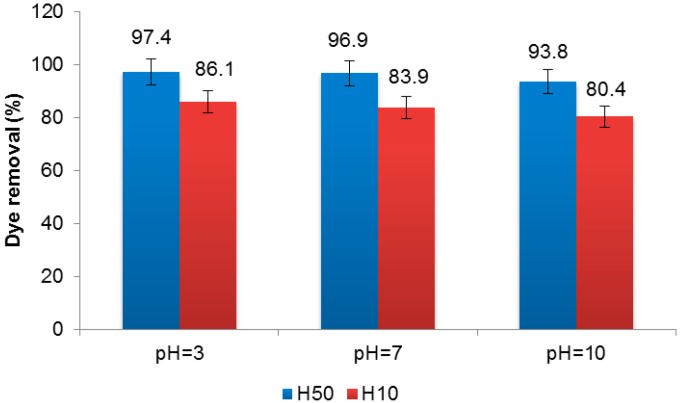
Effect of pH on CY dye removal by a nanofiltration membrane.

The pH can also affect the physical properties of dye solution. The experiments carried out at different pH values (3, 7, and 10), showed that the nanofiltration is efficient in all studied cases, although the highest efficiency was achieved at pH 3 for both membranes. With H50, 97.4% dye removal was obtained at pH 3 and 94% at pH 10 whereas, with H10, the yield was 86.1% at pH 3 and 80 at pH 10 (Figure 6).

The effect of pH on the nanofiltration process was also observed by other authors [34,35]. The pH can influence in both the agglomeration of dye particles and the hydrophobicity of the membrane.

**Figure 7:**
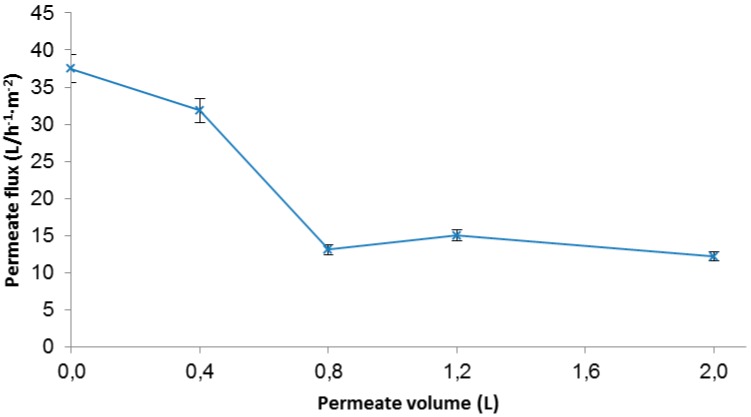
Effect of pH on CY dye removal by a nanofiltration membrane.

As it is known, industrial effluents from dyeing processes with reactive dyes contain high amounts of salt and have strong alkaline pH. The synthetic effluents tested at these conditions (60 g·L^−1^ NaCl and pH 10) showed colour removal higher than 80% for both membranes. From these results, it was decided that the H10 membrane exhibited enough colour removal for further study. In addition, the H10 membrane has a molecular weight cut-off higher than H50 and, therefore, could be less susceptible to experience fouling. In order to know the behaviour of the H10 membrane, a longer test was carried out. The permeate flux values are shown in Figure 7.

As can be observed in Figure 5, for the first 0.4 L the permeate flux decreased from 38 to 32 L/h^−1^·m^−2^. However, from this point, the permeate flux decreased up to 13 L/h^−1^·m^−2^, which represents a loss of 65%. Then, the permeability flux remained stable until the end of the experiment. After cleaning the membrane, the permeate flux was 29 L/h^−1^·m^−2^ (64.4% of the flux lost was recovered). Therefore, the fouling observed was mostly reversible.

### 3.2. Electrochemical Treatment

The synthetic dye baths containing CY and concentrated by the H10 membrane (1 g·L^−1^) were treated by means of electrochemical process. The effect of current density on dye removal efficiency was tested. Electrochemical treatment was performed at three current densities: 33, 83, and 166 mA/cm^2^ until 98% dye removal was observed.

The electrochemical treatment was able to degrade the azo group of the dye resulting in the solution colour removal. This is mainly due to the reaction of dye with the generated oxidant species, such as chlorine/hypochlorite, according to the reactions 4–6 [36]:

2Cl^−^ → Cl_2(aq)_ + 2e^−^ (4)

Cl_2(aq)_ + H_2_O → ClO^−^ + Cl^−^ + 2H^+^ (5)

Dye (C,H,O,N) + ClO^−^ → intermediate compounds → CO_2_ + H_2_O + N_2_ + Cl^−^ (6)

As is shown in Table 2, the colour removal efficiency was increased with increasing the applied current density. This is due to the increasing rate of generation of oxidants [37].

A linear relationship between the applied current density and time of treatment was observed. The selection of the operational current density will depend on the time available for the treatment and the power supply used.

Due to the linear relationship observed, it was expected that the power consumption was the same for the three current densities studied. However, power consumption increased with increasing the applied current density, which can be attributed to limitations of the power supply, which is more efficient when low intensities are used.

### 3.3. Effluent Reuse

The permeate and the concentrated effluents were used in order to study the feasibility of water reuse in new dyeing processes.

#### 3.3.1. Permeate Reuse

Although the H10 membrane was selected to be combined with the electrochemical treatment, the permeate reuse study was evaluated for both membranes. The dyeings were carried out with 100% of permeate from the H50 membrane (Table 3) and the H10 membrane (Table 4). Dyeings obtained were evaluated with respect to a reference dyed with softened tap water.

It can be observed that dyeings showed DE_CMC(2:1)_ values significantly lower than 0.5, the maximum DE_CMC(2:1)_ value accepted at the industrial scale being 1.5. In general, the values of DL were negative, which means that dyeings with permeate were darker than the reference. Therefore, lower dye concentration would be added to obtain the same intensity, which provides an important advantage from the economical point of view.

The DH and DC values did not follow a trend. In general, these values were low, being attributed to experimental limitations in the measurements. In the case of CY dye, the DC value was considerably higher than the other. It can be concluded that the residual dye in the permeate has more influence when new dyeings are carried out with the treated effluent containing the same dye.

#### 3.3.2. Reuse of the Decoloured Concentrate Effluent

Taking our previous studies into consideration, 70% of decoloured effluent was reused. Table 5 shows the results obtained in the reuse study of the decoloured concentrate All dyeings carried out showed a DE_CMC(2:1)_ higher than that obtained in the permeate reuse, which can be attributed to the higher content of residual organic matter. It is important to notice that the dye concentration in this effluent was 10 times higher than the dye concentration of effluents treated using nanofiltration membranes. The electrochemical treatment was only applied to remove colour, not to mineralize the organic matter. A complete mineralization could be obtained with longer treatment, which would imply an increase in the cost of the process. However, the decolourization of the effluent was sufficient to satisfy the established criteria (DE_CMC(2:1)_ < 1.5).

## 4. Conclusions

The nanofiltration treatment provided up to 98% dye removal with the H50 membrane and up to 86% with the H10 membrane. Regarding the membranes’ behaviour, no significant fouling was observed during the experiments. Although in a longer test, the H10 membrane showed 65% permeate flux loss, and the fouling observed was mostly reversible. The effect of salt concentration in the membrane rejection of CY was tested. The presence of NaCl did not affect the retention of the dye with the H50 membrane. In the case of H10 membrane, a significant increase of dye removal in the presence of salt was observed. The effect of pH was also studied showing that is not an important factor in the efficiency of nanofiltration membranes. The highest dye removal was achieved at pH 3, whereas the lowest was at pH 10.

The effluent concentrated with the H10 membrane was decoloured by means of an electrochemical treatment. The higher current density, the better dye the removal efficiency because of the greater generation of oxidizing species.

Finally, the combination of nanofiltration membranes and electrochemical process allowed reuse between 70% of the electrochemically decoloured effluent and 100% of permeate in new dyeing processes. It was also possible to reuse the salt present in the effluents with the subsequent economic savings. The results of the water reuse are very promising for the textile industry.

## Figures and Tables

**Table 1:** H50 and H10 specifications.

Membrane Characteristics	H50	H10
Membrane Polymer	Sulfonated Polyethersulfone	Sulfonated Polyethersulfone
Molecular Weight Cut-off	1000 daltons	3000 daltons
Maximum Applied Pressure	41 bar	41 bar
Maximum Continuous Chlorine Concentration	0.01 g·L^−1^	0.01 g·L^−1^
Maximum Chlorine Concentration for Cleaning	0.1 g·L^−1^	0.1 g·L^−1^
Maximum Operating Temperature	60 °C	60 °C
Operating pH Range	2–11	2–11
Cleaning pH Range	1–12	1–12

**Table 2:** Electrochemical degradation of CY and power consumption at three current densities.

Current Density (mA/cm^2^)	Intensity (A)	Voltage (V)	*t* (h)	Power Consumption (W·h·L^−1^)
33	2	4.5	10	45 ± 0.03
83	5	5.2	4	52 ± 0.02
166	10	6.3	2	63 ± 0.08

**Table 3:** Colour differences values for the H50 membrane.

	H50 Membrane			
Dye	DH	DL	DC	DE_CMC(2:1)_
CY	0.24 ± 0.04	−1.03 ± 0.05	0.46 ± 0.04	0.42 ± 0.02
PY	−0.74 ± 0.02	−0.74 ± 0.05	−0.07 ± 0.02	0.31 ± 0.02
PC	0.21 ± 0.03	−0.28 ± 0.03	0.16 ± 0.07	0.18 ± 0.03
PN	0.30 ± 0.02	−0.29 ± 0.02	0.02 ± 0.04	0.37 ± 0.04

**Table 4:** Colour differences values for the H10 membrane.

	H10 Membrane			
Dye	DH	DL	DC	DE_CMC(2:1)_
CY	−0.07 ± 0.02	−0.51 ± 0.01	2.16 ± 0.04	0.28 ± 0.03
PY	−0.40 ± 0.02	−0.80 ± 0.05	−0.44 ± 0.04	0.34 ± 0.02
PC	0.73 ± 0.06	−0.28 ± 0.04	0.11 ± 0.03	0.28 ± 0.02
PN	−0.08 ± 0.05	0.21 ± 0.05	−0.34 ± 0.02	0.25 ± 0.01

**Table 5:** Colour differences values for decoloured concentrate effluent reuse.

Dye	DH	DL	DC	DE_CMC(2:1)_
CY	−0.10 ± 0.04	0.46 ± 0.10	1.29 ± 0.07	1.39 ± 0.04
PY	0.30 ± 0.05	0.16 ± 0.04	−0.06 ± 0.04	0.87 ± 0.06
PC	0.24 ± 0.02	0.32 ± 0.05	−0.31 ± 0.02	0.32 ± 0.06
PN	0.17 ± 0.06	−0.18 ± 0.11	−0.16 ± 0.02	0.35 ± 0.09
